# Adolescent trauma and alcohol: increased risk for severe head injury and organ dysfunction–insights from the TraumaRegister DGU^®^

**DOI:** 10.1007/s00068-025-02993-6

**Published:** 2025-10-28

**Authors:** Jason-Alexander Hörauf, Jan-Niklas Franz, Ramona Sturm, Rolf Lefering, Borna Relja, Ingo Marzi, Nils Wagner, TraumaRegister DGU

**Affiliations:** 1 Department of Trauma Surgery and Orthopedics, University Hospital Frankfurt, Goethe-University, Frankfurt am Main, Germany; 2 Institute for Research in Operative Medicine (IFOM), University Witten/Herdecke, Cologne, Germany; 3 Department of Trauma, Hand, Plastic and Reconstructive Surgery, Translational and Experimental Trauma Research, University Ulm, Ulm, Germany; 4 Committee on Emergency Medicine, Intensive Care and Trauma Management (Sektion NIS) of the German Trauma Society (DGU), Berlin, Germany

**Keywords:** Alcohol, Trauma patients, Injury severity, Adolescent trauma

## Abstract

**Purpose:**

Alcohol consumption is common among young individuals and is a significant, yet preventable, risk factor for injuries and fatalities. In adults, it influences injury patterns, severity, and potentially outcomes via immunomodulatory effects. However, data in adolescents are scarce. This study seeks to address this deficiency by analyzing data from the TraumaRegister DGU^®^ (TR-DGU) to investigate the impact of alcohol on adolescent trauma patients.

**Methods:**

This retrospective TR-DGU analysis included trauma patients aged 10–20 years admitted between 2015 and 2019. Patients were stratified by blood alcohol level (BAL positive vs. negative) and analyzed for demographics, injury patterns, prehospital characteristics, and in-hospital outcomes, including ICU length of stay (LOS), organ failure, and mortality.

**Results:**

Of 1,823 patients, 1,481 were BAL- and 324 BAL+. Alcohol-positive incidence increased over the study period, particularly in older adolescents, and was more frequent in winter, on weekends, and at night. BAL + patients were more often involved in violent and penetrating trauma and sustained more severe head injuries (43.6% vs. 36.5%, *p* = 0.016) but were intubated less often (30.8% vs. 34.5%, *p* = 0.099), significantly so in the 18–20-year group. BAL + adolescents had higher rates of respiratory organ failure (15.2% vs. 8.3%, *p* = 0.014) and sepsis (5.9% vs. 2.8%, *p* = 0.03), with no significant differences in ICU LOS or mortality.

**Conclusion:**

Alcohol use remains a significant factor among adolescent trauma patients in German-speaking countries. As trauma mechanisms vary by age, targeted prevention strategies are crucial. Intoxicated adolescent trauma patients form a high-risk group requiring special attention. Further research into alcohol’s immunomodulatory effects in this population is essential to improve trauma care strategies.

## Introduction

Traumatic injuries are a leading cause of morbidity and mortality among adolescents [1, 2]. Globally, alcohol is a major contributor to disease and injury, with nearly half of all alcohol-related deaths resulting from unintentional or violent injuries [3]. Estimates suggest that over 30% of all patient visits to trauma centers are alcohol-related, highlighting the significant impact of alcohol consumption in trauma care [4]. Furthermore, alcohol consumption appears to influence the clinical outcome of trauma patients. However, recent studies have primarily focused on its impact on injury severity and outcomes in adults, yet the available evidence remains inconclusive [5, 6]. While some studies suggest that adult patients with positive blood alcohol levels sustain more severe injuries and have higher mortality rates, others report no significant differences or even indicate potential protective effects of alcohol intoxication compared to patients without detectable alcohol levels [7–11].

The 2019 report of the European School Survey Project on Alcohol and Other Drugs (ESPAD) highlights the continued prevalence of alcohol abuse among adolescents in Europe, particularly in Germany. Among surveyed German students, 38% reported alcohol use by age 13 or younger, and 54% reported heavy episodic drinking within the past 30 days [12]. These rates are comparable to those observed in other Western countries, as shown by the Health Behavior in School-Aged Children (HBSC) study and the U.S. Substance Abuse Survey [13]. Although the issue of adolescent alcohol consumption - particularly binge drinking - has been recognized for years, specific epidemiological data on its impact on trauma characteristics and clinical outcomes in this population remain limited. This lack of data is primarily due to the fact that most studies predominantly include patients over the age of 18, leaving a substantial part of the adolescent population unrepresented. Adolescents and young adults are particularly vulnerable, as this stage of life is characterized by both increased alcohol consumption and a heightened tendency toward risky behaviors [14]. Additionally, alcohol consumption is a significant risk factor for injury, as it impairs motor skills, increases risk-taking behaviors, and slows reaction times [15–17]. Adolescents differ from adults in developmental stage, drinking patterns, and risk-taking behavior, yet this group has received comparatively little attention in trauma research. Consequently, it remains uncertain to what extent the conflicting associations between alcohol and trauma outcomes described in adults also apply to younger patients.

Since adolescent alcohol use is widely socially accepted in Western societies, there is an urgent need for research to develop effective preventive strategies and clinical care. This study aims to address this gap by utilizing data from the largest German trauma database TraumaRegister DGU^®^ (TR-DGU) to provide epidemiological insights into alcohol-intoxicated adolescent trauma patients and to assess the potential impact of alcohol consumption on clinically relevant outcome parameters.

## Methods

### TraumaRegister DGU^®^

The retrospective study was performed using data from the TraumaRegister DGU^®^ (TR-DGU) of the German Trauma Society (Deutsche Gesellschaft für Unfallchirurgie, DGU). This registry was founded in 1993. The aim of this multi-center database is a pseudonymized and standardized documentation of severely injured patients. Data are collected prospectively in four consecutive time phases from the site of the accident until discharge from the hospital: (A) Prehospital phase, (B) Emergency room (ER) and initial surgery, (C) Intensive care unit (ICU), and (D) Discharge. The documentation includes detailed information on demographics, injury patterns, comorbidities, pre- and in-hospital management, course on ICU, and relevant laboratory findings including data on transfusion and outcome of each individual. The inclusion criterion is admission to the hospital via ER with subsequent ICU care or reaching the hospital with vital signs and dying before admission to the ICU.

The infrastructure for documentation, data management, and data analysis is provided by AUC - Academy for Trauma Surgery (AUC - Akademie der Unfallchirurgie GmbH), a company affiliated to the German Trauma Society. The scientific leadership is provided by the Committee on Emergency Medicine, Intensive Care, and Trauma Management (Sektion NIS) of the German Trauma Society. The participating hospitals submit their data pseudonymized into a central database via a web-based application. Scientific data analysis is approved according to a peer review procedure laid down in the publication guideline of TR-DGU.

The participating hospitals are primarily located in Germany (90%), but a rising number of hospitals from other countries contribute data as well (at the moment from Austria, Belgium, Finland, Luxembourg, Slovenia, Switzerland, The Netherlands, and the United Arab Emirates). Currently, about 28,000 cases from almost 700 hospitals are entered into the database per year. Participation in TR-DGU is voluntary. For hospitals associated with TraumaNetzwerk DGU^®^, however, the entry of at least a basic data set is obligatory for reasons of quality assurance.

Scientific data analysis is conducted following an internal peer review process within the registry. The present study complies with the publication guidelines of TR-DGU and is registered under project ID 2020–019.

### Study cohort and parameters

Since the parameter „alcohol“ was incorporated into the TR-DGU dataset in 2015, all patients documented from that point onward were potentially eligible for inclusion. However, documentation of alcohol levels is not mandatory and is only reported if tested; therefore, not all documented patients were available for analysis. Additionally, patients transferred from another hospital (without an initial measurement available) and those transferred early (< 48 h) to a different hospital (without a final outcome available) were excluded from the study.

**Fig. 1:**
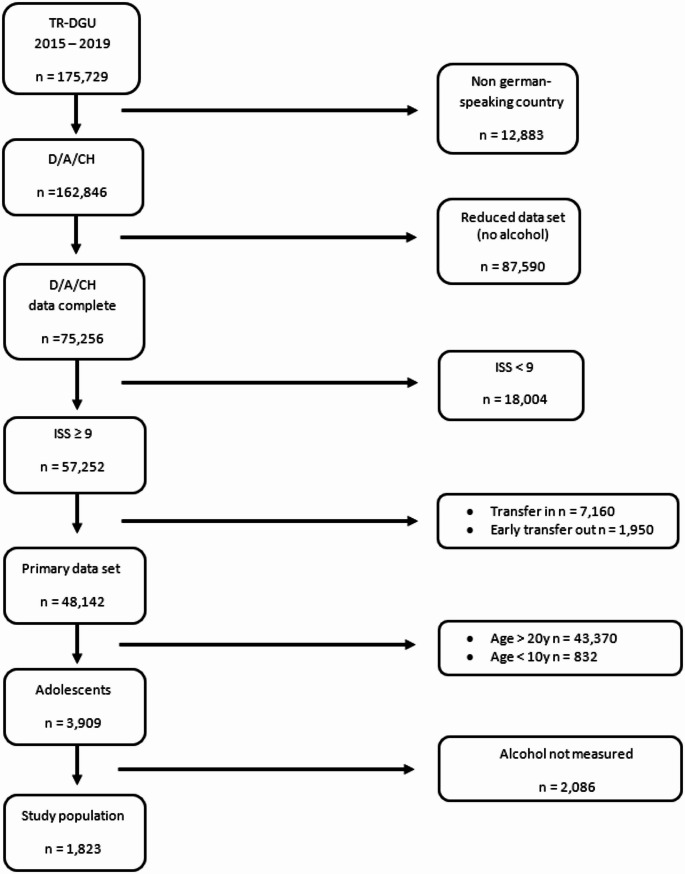
Flowchart study population

According to the World Health Organization (WHO), adolescence is defined as “the phase of life between childhood and adulthood, [.] a unique stage of human development and an important time for laying the foundations of good health [18].” Based on this definition, we included patients aged 10 to 20 years of age in our study. The detailed selection process is depicted in Fig. 1.

For analysis, adolescent trauma patients were categorized into two subgroups based on their blood alcohol level (BAL): BAL-negative (BAL-) and BAL-positive (BAL+).

Various parameters, including prehosiptal, in-hospital, and outcome variables, as well as epidemiological factors, trauma mechanisms, and accident circumstances, were compared between the groups.

Injury severity was assessed using the Injury Severity Score (ISS) developed by Baker et al. [19], with an ISS ≥ 9 considered clinically relevant. The TR-DGU defines organ failure based on the Sequential Organ Failure Assessment (SOFA) score, as described by Vincent et al. [20]. Organ failure is defined as a SOFA score of 3 or 4 points sustained for at least two consecutive days. Multiple organ failure is defined as concurrent failure of at least two organs, while sepsis is diagnosed when at least two systemic inflammatory response syndrome (SIRS) criteria are met al.ongside a positive blood culture. The Revised Injury Severity Classification II (RISC II) score has been developed and validated for mortality prediction depending on the clinical status in the emergency room with a large number of patients from TR-DGU. The RISC II score incorporates factors such as the Abbreviated Injury Scale (AIS) severity of the most and second-most severe injuries, head injury, age, sex, pupil response, preinjury health status, blood pressure, acidosis, coagulation, hemoglobin levels, and the necessity of cardiopulmonary resuscitation (CPR). The RISC II score (where a lower value indicates better survival prospects) is subsequently transformed into a risk of death estimate using a logistic function [21].

### Statistical analysis

Statistical analysis was performed using SPSS statistical software (version 26, IBM Inc., Armonk, NY, USA). Continuous variables are presented as mean ± standard deviation (SD), while categorical variables are expressed as numbers and/or percentages. For skewed data, the median with interquartile range (IQR) was reported instead of the mean and SD. Group differences were assessed using the Mann-Whitney U-test for continuous variables and Pearson’s Chi-squared test or Fisher’s exact test, as appropriate, for categorical variables. A significance level of 0.05 applied. The figures were created using GraphPad Prism (version 9.4.0 GraphPad Software Inc. San Diego, CA, USA).

## Results

In total, 57,252 patients with an ISS ≥ 9 were documented between 2015 and 2019 in hospitals across Germany, Austria, and Switzerland. Of these, 55,398 patients were excluded based on the predefined exclusion criteria outlined in detail in Fig. 1. Consequently, 1,823 patients were included in the final analysis.

A total of 1,481 adolescent patients tested negative for blood alcohol on hospital admission (BAL-, 81.2%), while 342 (18.8%) patients had positive blood alcohol levels (BAL+). Patients in the BAL + group were more frequently male (84.5% vs. 69.9% in the BAL- group, *p* < 0.001) and slightly older (18 ± 2.5 years BAL + vs. 17.1 ± 1.9 years BAL-, *p* < 0.001). The mean blood alcohol concentration (BAC) in the BAL + group was 0.8 ± 0.88‰. BAC increased with age, averaging 0.21 ± 0.30 g/L in 10- to 14-year-olds, 0.70 ± 0.85 in 15- to 17-year-olds, and 0.88 ± 0.9 in the 18- to 20-year-olds.

No significant differences were observed in ICU stay, or ISS. Regarding injury patterns by body region, alcohol-intoxicated adolescents had a significantly higher proportion of severe injuries (AIS ≥ 3) to the head (36.5% BAL- vs. 43.6%, BAL+, *p* = 0.016) and face (5.1% BAL- vs. 9.9%, BAL+, *p* < 0.001), while severe pelvic injuries were less frequent (12.4% BAL- vs. 7.9% BAL+, *p* = 0.019). A trend toward fewer severe extremity injuries was noted (39.7% BAL- vs. 34.2% BAL+, *p* = 0.055). No significant differences were found for chest or abdominal injuries. Table 1 summarizes patient characteristics and injury patterns.

**Table 1:** Summary of patient characteristics and injury pattern

Patient Characteristics	BAL- (*n* = 1481)	BAL+ (*n* = 342)	*p*-value
Male sex (n, %)	1,035 (69.9%)	289 (84.5%)	**< 0.001**
Age (years ± SD)	17.1 ± 2.5	18.0 ± 1.9	**< 0.001**
Days in hospital (median, IQR)	12 (6–20)	11 (6–19)	0.64
Days on ICU (median, IQR)	3 (1–8)	2 (1–8)	0.45
BAC total (‰ mean ± SD)	0.00	0.8 ± 0.88	
BAC 10–14 years (g/L mean ± SD)	0.00	0.21 ± 0.30	
BAC 15–17 years (g/L mean ± SD)	0.00	0.70 ± 0.85	
BAC 18–20 years (g/L mean ± SD)	0.00	0.88 ± 0.90	
ISS	20.8 ± 12.5	20.4 ± 12.0	0.80
Severely Injured body regions (AIS ≥ 3)			
Head (n, %)	540 (36.5%)	149 (43.6%)	**0.016**
Face (n, %)	76 (5.1%)	34 (9.9%)	**0.001**
Chest (n, %)	544 (36.7%)	113 (33.0%)	0.21
Abdomen (n, %)	241 (16.3%)	50 (14.6%)	0.51
Spine (n, %)	149 (10.1%)	32 (9.4%)	0.76
Extremities (n, %)	588 (39.7%)	117 (34.2%)	0.055
Pelvis (n, %)	183 (12.4%)	27 (7.9%)	**0.019**

Data are presented as mean ± standard deviation (SD) or as n (%). *P*-values refer tocomparisons between BAL+ vs. BAL- groups. Note that some data were not available forall cases

Abbreviations BAL- group= negative blood alcohol concentration, BAL+ group= bloodalcohol concentration ≥ 0.1‰;*AIS=* Abbreviated Injury Scale, *ICU=* Intensive Care Unit, *IQR=* Interquartile Range, *ISS=*Injury Severity Score, *SD=* Standard Deviation

**Fig. 2:**
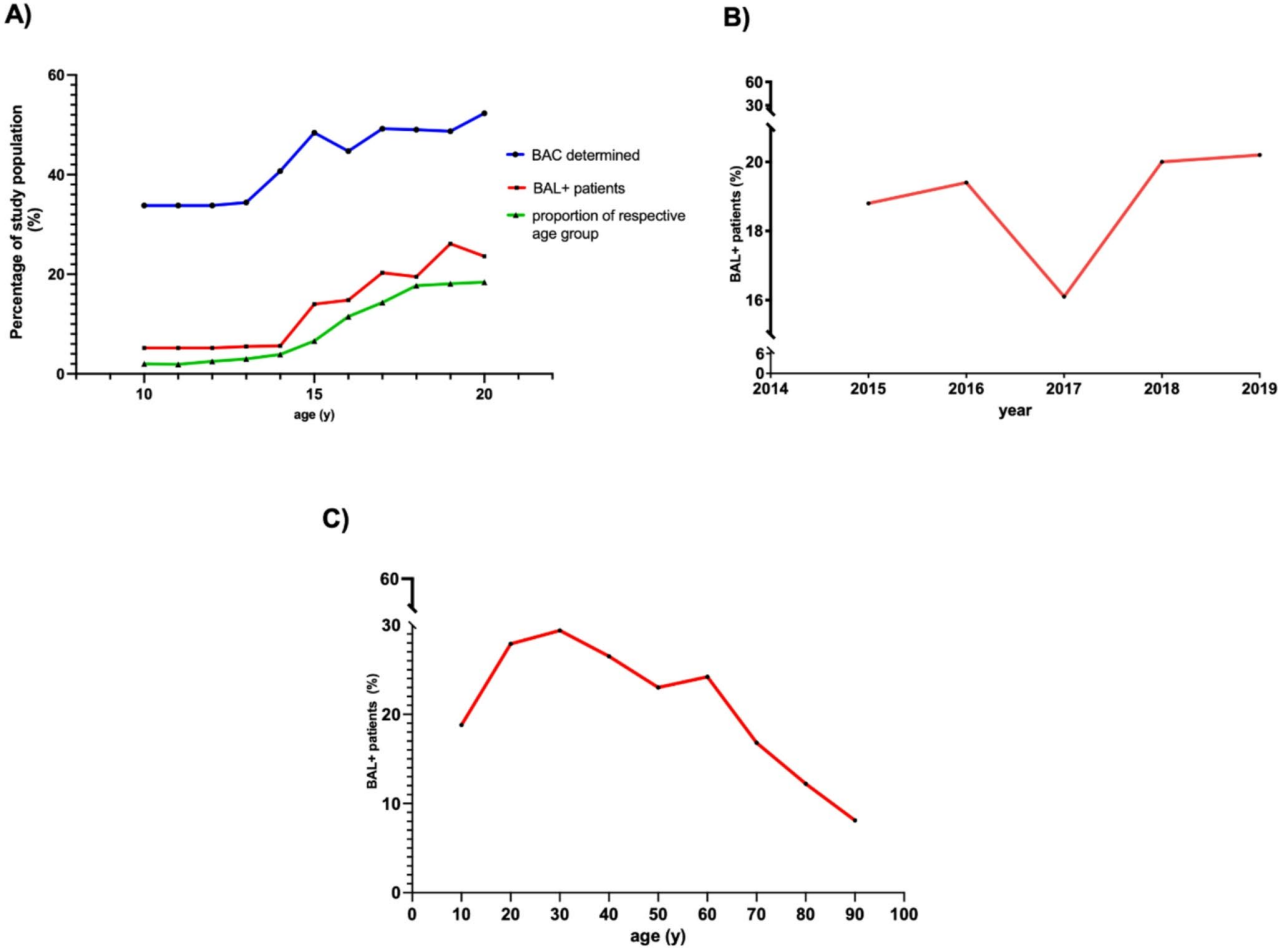
Epidemiology Epidemiological characteristics of the study population are shown. Alcohol detection rates and the proportion of alcohol-intoxicated trauma patients increased with age **A**: Proportion of patients in each age group with blood alcohol level (BAL) testing (blue), proportion of BAL + patients within each age group (red), and the relative contribution of each age group to the total study population (green). **B**: Temporal trend in the proportion of BAL + patients over the study period. **C**: Proportion of BAL + patients stratified by age decade

In our study cohort, alcohol detection rates and the proportion of alcohol-intoxicated trauma patients (TP) increased with age (Fig. 2A). Older patients also constituted a larger proportion of the study population (Fig. 2A). Except for 2017, the percentage of BAL + TP showed a continuous rise over time (Fig. 2B). When categorized by age group and severity of intoxication, the proportion of both sub severely (0‰ ≤ BAC ≤ 2‰) and severely (BAC ≥ 2‰) intoxicated individuals increases significantly with age. Among 10- to 14-year-olds, 94.7% had a BAC of 0‰, while 5.3% were mildly intoxicated, and none had a BAC ≥ 2‰. In the 15- to 17-year-old group, 82.9% had a BAC of 0‰, 15.4% were mildly intoxicated, and none were severely intoxicated. Among 18- to 20-year-olds, 76.9% had a BAC of 0‰, 20.2% were mildly intoxicated, and 2.8% had a BAC ≥ 2‰.

Stratified by time of day and week, alcohol-intoxicated patients were significantly more frequent on weekends than from Monday to Thursday (36.8% vs. 63.2%, *p* < 0.001) and more common at night than during the day (69.0% vs. 31.0%, *p* < 0.001). Regarding seasonal distribution, the highest proportion of alcohol-intoxicated adolescents was treated in winter and the lowest in summer (22.2% vs. 16.5%, *p* = 0.046). There was no significant seasonal variation in the distribution of BAL + compared to BAL- trauma patients (Table 2).

**Table 2:** Temporal, seasonal, and local incidence

Incidence	BAL- (*n* = 1481)	BAL+ (*n* = 342)	*p*-value
Trauma center level of care supra-regional (*n*, %) regional (*n*, %) local (*n*, %)	1,293 (87.3%) 167 (11.3%) 21 (1.4%)	308 (90.1%) 33 (9.6%) 1 (0.3%)	0.148
Daytime (06:00am-5:59pm) (n, %) Nighttime (06:00pm-05:59am) (n, %)	777 (52.5%) 704 (47.5%)	106 (31.0%) 236 (69.0%)	**< 0.001**
Week (Monday-Thursday, n, %) Weekend (Friday-Sunday, n, %)	767 (51.8%) 714 (48.2%)	126 (36.8%) 216 (63.2%)	**< 0.001**
Winter (n, %)	246 (16.6%)	70 (20.5%)	0.105
Spring (n, %)	380 (25.7%)	96 (28.0%)	0.397
Summer (n, %)	474 (32.0%)	94 (27.5%)	0.118
Fall (n, %)	381 (25.7%)	82 (24.0%)	0.548

Data are presented as n and (%). *P*-values refer to comparisons between BAL + vs. BAL- groups. Note that some data were not available for all cases

*Abbreviations* AIS = Abbreviated Injury Scale, *BAL*- group = negative blood alcohol concentration, *BAL* + group = blood alcohol concentration ≥ 0.1‰, *BPsys* = Systolic blood pressure, *ED* = Emergency Department, *GCS* = Glasgow Coma Scale, *MOF* = Multi Organ Failure, *OF* = Organ Failure, *RISC II* = Revised Injury Severity Classification II, *SD* = Standard Deviation

Alcohol-intoxicated patients were more frequently admitted to superregional trauma centers (BAL- 87.3.% vs. BAL + 90.1%) and less often to regional and local trauma centers, however, this trend was not statistically significant.

Regarding trauma mechanisms, alcohol-intoxicated trauma patients were significantly more likely to sustain injuries from violent incidents than their non-intoxicated counterparts (8.0% BAL + vs. 2.2% BAL-, *p* < 0.001). However, no significant difference was observed for trauma related to suicidal intent (4.8% BAL- vs. 5.0% BAL+, *p* = 0.88). Our data indicate a disproportionate increase in alcohol-intoxicated TP with penetrating injuries compared to those with blunt trauma as age increases. Among 10- to 14-year-olds, alcoholized adolescents were more common in blunt trauma cases than in penetrating trauma (5.2% BAL + TP in blunt trauma vs. 0.0% BAL + TP in penetrating trauma). However, this trend reversed in older age groups, with a notable increase of alcoholized adolescents sustaining penetrating trauma among the 15- to 17-year-olds (30.4% BAL + TP in penetrating trauma vs. 16.5% BAL + TP in blunt trauma) and 18- to 20-year-olds (39.6% BAL + TP in penetrating trauma vs. 22.0% BAL + TP in blunt trauma) (Table 3).

**Table 3:** Summary prehospital therapy and outcome

Prehospital interventions	BAL-(n = 1481)	BAL+(n = 342)	p-value
Prehospital intubation (n,%)	506 (34.5%)	103 (30.8%)	0.099
Fluid administration preclinical (Lmean ± SD)	0.86 ± 0.63	0.85 ± 0.58	0.84
Fluid administration in the ED (L mean ± SD)	1.42 ± 1.90	1.40 ± 1.73	0.71
Shock, BPsys ≤ 90 preclinical (n, %)	136 (10.1%)	32 (10.3%)	0.92
GCS ≤ 13 prehospital (n, %)	580 (39.8%)	160 (48.0%)	**0.007**
GCS ≤ 8 prehospital (n, %)	313 (22.1%)	86 (26.7%)	**0.043**
Within GCS ≤ 8			
Relevant head injury (AIS≥ 3)	264 (84.3%)	73 (84.9%)	0.90
No relevant head injury	49 (15.7%)	13 (15.1%)	
GCS ≤ 8 without AIS head ≥3	49 (3.5%)	13 (4.0%)	0.36
Trauma mechanism			
Car (n, %)	433 (29.3%)	111 (32.6%)	0.23
Motorcycle (n, %)	388 (26.3%)	51 (15.0%)	**<0.001**
Bicycle (n, %)	126 (8.5%)	22 (6.5%)	0.18
Pedestrian (n, %)	119 (8.1%)	36 (10.6%)	0.16
High Fall (n, %)	187 (12.7%)	60 (17.6%)	**0.026**
Low Fall (n, %)	76 (5.1%)	18 (5.3%)	0.91
Others (n, %)	149 (10.1%)	42 (12.4%)	0.24
Blunt trauma (n, %)	1,389 (96.5%)	303 (92.1%)	**<0.001**
Violence (n, %)	32 (2.2%)	27 (8.0%)	**<0.001**
Suicide (n, %)	71 (4.8%)	17 (5.0%)	0.88
Organ failure			
Lung (n, %)	114 (8.3%)	47 (15.2%)	**0.014**
Blood/Coagulation (n, %)	75 (5.4%)	23 (7.4%)	0.22
Liver (n, %)	7 (0.5%)	5 (1.6%)	0.14
Heart/Circulation (n, %)	250 (18.1%)	68 (21.9%)	0.14
Central nervous system (n, %)	223 (16.1%)	63 (20.3%)	**0.094**
Kidney (n, %)	27 (2.0%)	8 (2.6%)	0.52
Sepsis (n, %)	38 (2.8%)	18 (5.9%)	**0.0**
MOF (n, %)	211 (15.3%)	60 (19.4%)	0.095
Mortality in hospital (n, %)	98 (6.6%)	21 (6.1%)	0.81
RISC II (%)	7.5%	6.9%	0.342

Data are presented as n and (%).*P*-values refer to comparisons between BAL+ vs. BAL- groups. Note that some data were not available for all cases

*Abbreviations* AIS= Abbreviated Injury Scale, BAL- group= negative blood alcohol concentration, BAL+ group= blood alcohol concentration ≥ 0.1‰, *BPsys*= Systolic blood pressure, *ED*= Emergency Department, *GCS*= Glasgow Coma Scale, *MOF*= Multi Organ Failure, *OF*= Organ Failure, *RISC II*= Revised Injury Severity Classification II, *SD*= Standard Deviation

Comparing accident causes between BAL- and BAL + groups, the most notable differences were observed in motorcycle-related road traffic accidents (RTA) and high falls. Motorcyclists accounted for 26.3% of BAL- TP but only 15.0% of BAL + TP (*p* < 0.001). In contrast, high falls were significantly more frequent in the BAL + group (17.6% vs. 12.7% BAL-, *p* = 0.026) (Table 3).

**Fig. 3:**
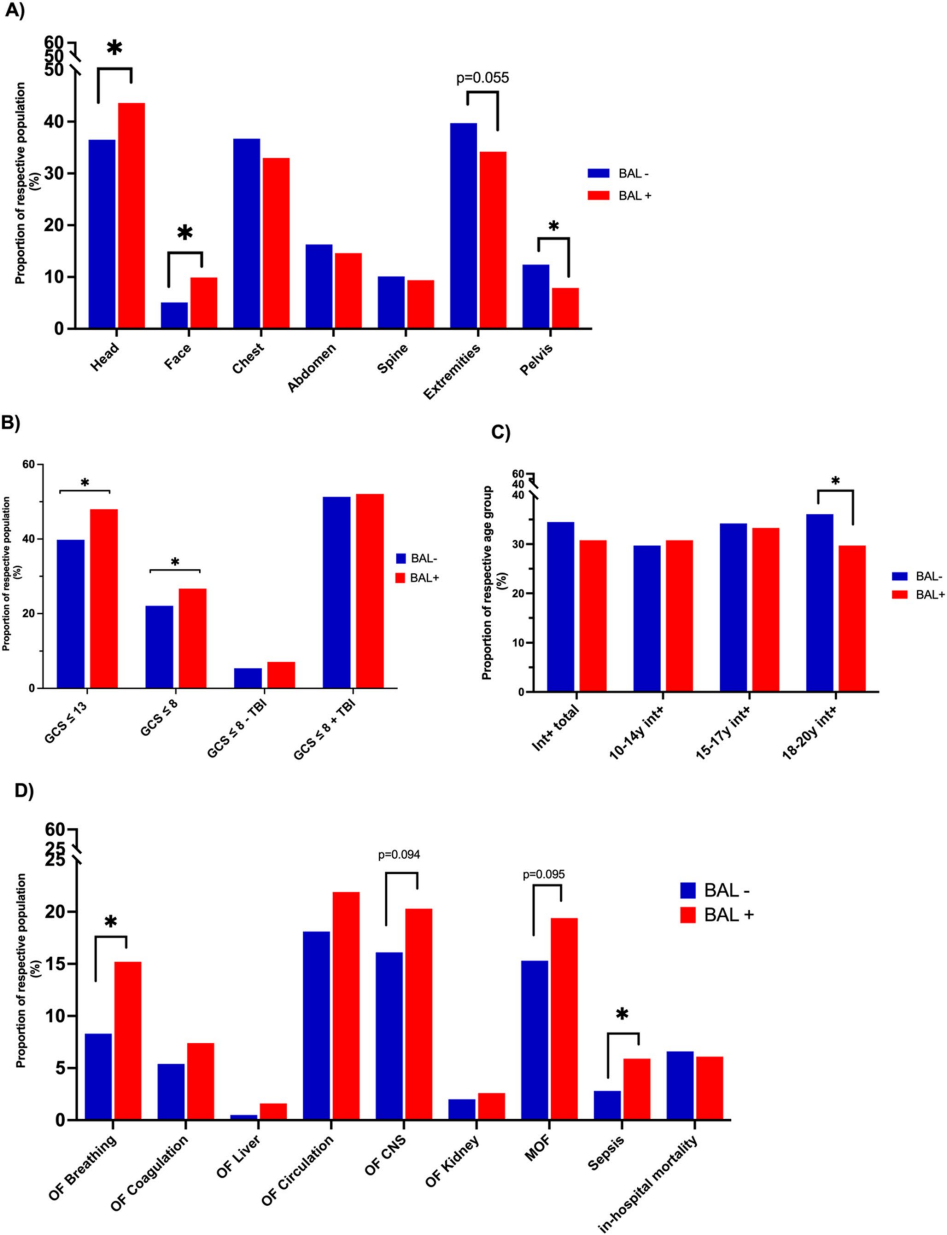
Prehospital characteristics, in-hospital complications, and outcome Selected prehospital characteristics and outcome parameters are shown. BAL + adolescents were significantly more often injured in the head and face region. Despite lower prehospital GCS scores, they were intubated less frequently than BAL- patients. Moreover, BAL + patients showed increased rates of organ failure and sepsis, although mortality was not higher **A**: Proportion of TP with severe injuries (AIS ≥ 3) in each anatomical region. **B**: Percentage of BAL- and BAL + TP presenting with a prehospital GCS ≤ 13 or ≤ 8, further subdivided by the presence or absence of TBI. **C**: Prehospital intubation rates, stratified by age group, comparing BAL- and BAL + TP. **D**: Incidence of organ failure, sepsis, and in-hospital mortality during the clinical course in BAL- versus BAL + TP

To investigate potential differences between BAL + TP and BAL- TP, as well as their impact on patient management and clinical outcomes, we additionally analyzed selected prehospital characteristics, in-hospital complications, and outcome parameters. BAL + TP sustained significantly more severe (defined as AIS ≥ 3) head (43.6% vs. 36.5% in BAL- TP; *p* = 0.016) and facial (9.9% vs. 5.9% in BAL- TP; *p* = 0.001) injuries compared to BAL- TP. Conversely, severe pelvic injuries were significantly less common in the BAL + group (7.9% vs. 12.4%; *p* = 0.019), accompanied by a trend toward a lower incidence of severe extremity injuries compared to the BAL- group (34.2% vs. 39.7%; *p* = 0.055) (Fig. 3A; Table 1). Additionally, the comparison between BAL- and BAL + TP showed a significantly higher prevalence of unconscious (defined as Glasgow Coma Scale ≤ 8) patients in the BAL + cohort (26.7% vs. 22.1% in BAL- TP, *p* = 0.043) (Fig. 3B). To assess whether impaired consciousness (GCS ≤ 8) was more attributable to alcohol intoxication or to concomitant TBI, we stratified patients by the presence or absence of severe head injury (defined as AIS_head_ ≥ 3) and compared the distribution between BAL + and BAL − groups. In the subgroup analysis of patients with a GCS ≤ 8, no significant difference in TBI prevalence was found between the BAL + and BAL- groups (Fig. 3B; Table 3). These findings suggest that the increased incidence of GCS ≤ 8 among BAL + patients may be attributed, at least in part, to the independent effects of alcohol intoxication on consciousness, rather than to a higher prevalence of severe traumatic brain injury. Although the GCS was significantly lower in the BAL + group, TP in this cohort were intubated less frequently (30.8% vs. 34.5% in BAL- TP; *p* = 0.099).

When examining organ failure (OF) and mortality, the BAL + group exhibited a notable trend towards higher rates of central nervous system (CNS) failure (20.3% vs. 16.1% in BAL- TP; *p* = 0.094) and multiple organ failure (MOF) (19.4% vs. 15.3% in BAL- TP; *p* = 0.095). Respiratory failure (15.2% vs. 8.3% in BAL- TP; *p* = 0.014) and sepsis (5.9% vs. 2.8% in BAL- TP; *p* = 0.03) were significantly more frequent in alcohol-intoxicated TP. However, no significant differences were observed in in-hospital mortality or RISC II scores (Table 3).

## Discussion

As alcohol consumption remains widespread among adolescents and current data on alcohol-intoxicated adolescent trauma patients are limited, the aim of this study was to analyze patient characteristics, injury patterns, and specific risk behaviors in this patient cohort in German-speaking countries. Given that acute alcohol intoxication may influence the clinical course and outcomes in adult trauma patients, these aspects were also examined in the present study.

Notably, the present study demonstrates a steady increase in the proportion of alcohol-intoxicated adolescent trauma patients between 2015 and 2019, surpassing 20% by the end of the study period. Moreover, the severity of intoxication, as indicated by blood alcohol concentration, was found to increase with age. However, the actual number of alcohol-intoxicated adolescent trauma patients may be higher than reported, as blood alcohol levels were assessed in only 52% of severely injured trauma patients even within the 18- to 20-year-old subgroup, highlighting the need for more comprehensive and standardized laboratory diagnostics, increased awareness of alcohol intoxication, and its potential adverse effects on the clinical course [22]. Moreover, our data suggest that prevention work needs to be intensified and awareness of the pronounced negative effects of alcohol on road traffic has to be strengthened.

In the analyzed cohort, trauma patients in the BAL + group presented significantly more often due to violence-related incidents compared to their non-intoxicated counterparts. This was accompanied by a significantly higher rate of penetrating injuries in the BAL + group (7.9%), exceeding the national average of 4–5% reported for severely injured patients in Germany [23]. Consistent with these findings, recently Verboket et al. revealed that intoxicated trauma patients account for the majority of violence-related incidents in a German urban university hospital [24]. Notably, the incidence of such cases rises during evening and nighttime hours, with intoxicated individuals accounting for up to 55% of all violent incidents [25]. Similarly, here we observed an increased incidence of alcohol-intoxicated adolescent trauma patients during evening and nighttime hours, with an even more pronounced peak on weekends. This constitutes a major challenge for in-hospital management and care, as during these times most EDs often operate with reduced staffing and limited resources [26]. Furthermore, our data show that the highest incidence of alcohol-intoxicated adolescent trauma patients occurs in supra-regional trauma centers. This has notable economic implications, as these centers are required to provide continuous, interdisciplinary care for severely injured patients [27, 28]. Beyond the direct costs of extended diagnostics, monitoring, and potentially prolonged ICU stays, alcohol-related trauma has been shown to increase overall healthcare expenditures and resource utilization, placing additional strain on high-level trauma centers [29].

Analysis of prehospital and in-hospital characteristics in our study cohort revealed seemingly contradictory findings. BAL + adolescent trauma patients were significantly more likely to sustain severe traumatic brain injuries (AIS_head_ ≥ 3) and to present with impaired consciousness (GCS ≤ 8) in the prehospital setting. Despite these indicators of severe neurologic compromise, BAL + patients were intubated less frequently prior to hospital admission. Recently, Rundhaug et al. demonstrated a dose-dependent association between alcohol intoxication and decreased GCS scores [30]. In our cohort, severe TBI was - as expected - strongly associated with impaired consciousness (GCS ≤ 8). However, among unconscious patients, no significant difference in the prevalence of severe TBI was observed between BAL + and BAL- groups. These findings suggest that the increased rate of reduced consciousness in the BAL + group cannot be solely explained by a higher incidence of TBI, but may also reflect the independent sedative effects of alcohol intoxication. Given these findings, it becomes all the more important to closely focus on the adequacy and safety of airway management in both BAL- and BAL + groups, as timely and appropriate airway protection is critical in TBI to prevent secondary neurological damage and poor outcomes [31]. Our analysis revealed a lower rate of prehospital intubation among BAL + patients, reaching statistical significance in the 18- to 20-year-old subgroup. This is particularly concerning, as a GCS ≤ 8 remains a well-established indication for emergency intubation in both adult and pediatric trauma care [32]. Despite similar injury patterns, severity, and shock rates, the lower intubation frequency in BAL + patients - who more often sustained severe TBI - raises concerns about underrecognition of critical neurological impairment. Given the adolescent CNS’s heightened vulnerability to secondary insults such as hypoxia and hypercapnia [33], inadequate airway protection may contribute to worse outcomes. This underlines the need for increased vigilance in the prehospital setting, particularly when managing intoxicated adolescents. Structured training emphasizing the differentiation between alcohol intoxication and TBI has been shown to improve recognition of clinical signs and may reduce the risk of misinterpretation in acute care situations [34]. However, despite the clinical relevance of this issue, recent surveys indicate that most hospitals lack systematic strategies or specific guidelines for the management of patients with TBI and concomitant alcohol intoxication in the acute phase [35]. These findings highlight the importance of developing standardized protocols together with targeted training for prehospital and in-hospital teams to minimize under-triage while optimizing early airway management in this vulnerable patient group.

Our findings reveal a significant increase in both respiratory organ failure and sepsis among BAL + patients, indicating a greater susceptibility to these complications in this subgroup. In line with our findings, Afshar et al. demonstrated that in severely injured trauma patients aged 16 to 21 years, higher blood alcohol concentrations were associated with an increased risk of developing acute respiratory distress syndrome (ARDS) [36]. Acute alcohol intoxication is known to impair respiratory center function in the medulla oblongata in a dose-dependent manner, leading to hypoventilation and bradypnea [37, 38]. These effects may be more pronounced in children and adolescents, who are less physiologically accustomed to ethanol exposure. Langhan et al. demonstrated hypoventilation episodes in up to 28% of intoxicated (non-trauma) adolescents, with significant oxygen desaturation occurring at BAC as low as 104 mg/dL [37]. Additionally, with up to 68% of adolescents experiencing vomiting within the first five hours in the ED, aspiration pneumonia must be considered a relevant contributor to respiratory failure and sepsis risk. Moreover, Lamminpää et al. reported respiratory acidosis in 32% of alcohol-intoxicated adult trauma patients, correlating inversely with BAC and directly with decreasing consciousness [39]. Experimental models support these findings, showing impaired metabolic and respiratory compensation in intoxicated animals following blunt chest trauma and hemorrhagic shock, leading to reduced physiological resilience and increased mortality [40]. These data underscore the importance of early airway management in intoxicated patients.

Beyond its neurological and respiratory effects, acute alcohol intoxication may also alter immune function [41–43]. It induces a shift toward a pro-inflammatory monocyte phenotype with functional suppression [44], reduces production of reactive oxygen species (ROS), and increases leukocyte apoptosis [45]. Experimental animal studies suggest that acute alcohol intoxication increases susceptibility to infection and sepsis by impairing neutrophil recruitment, neutrophil extracellular traps, and macrophage function, leading to reduced bacterial clearance and delayed infection resolution [46, 47]. Collectively, these findings indicate that ethanol exposure can disrupt innate immune responses and compromise host defense against pathogens.

Although increased rates of organ failure and sepsis in BAL + adolescents were not accompanied by higher in-hospital mortality or prolonged ICU length of stay in our cohort, these complications remain clinically highly relevant. Both organ dysfunction and infectious complications are known to increase the need for rehabilitation and may contribute to long-term morbidity and impaired quality of life [48, 49]. Moreover, prolonged antibiotic therapy carries the risk of adverse effects and resistance development that can further compromise recovery and negatively affect long-term outcomes [50]. While registry data are not designed to capture such outcomes, it is reasonable to assume that alcohol-intoxicated adolescents experiencing these complications face a more complex recovery trajectory with greater resource utilization.

This study highlights distinct clinical and epidemiological characteristics associated with alcohol-intoxicated adolescent trauma patients. Compared to their non-intoxicated counterparts, BAL + patients were significantly more likely to be male and to sustain severe head and facial injuries, accompanied by lower GCS scores upon presentation. Notably, despite these indicators of neurologic compromise, the 18- to 20-year-old BAL + subgroup was intubated significantly less frequently in the prehospital setting. Furthermore, BAL + patients were more often admitted during nighttime hours and weekends and were disproportionately involved in violence-related incidents and penetrating trauma. Clinically, they experienced higher rates of respiratory failure and sepsis during the hospital course. However, these complications did not translate into increased in-hospital mortality. In comparison with the adult population, the primary issues—particularly TBI and oral and maxillofacial injuries—are likewise predominant among adolescents. This suggests that the key challenges in managing alcohol-intoxicated trauma patients remain consistent across age groups. Nonetheless, differences in pathophysiology and clinical course may exist, as adolescents could respond differently to alcohol and trauma due to their ongoing physical and immunological development. If, however, it is confirmed that care priorities and major complications in adolescents closely mirror those in adults, this would support the direct application of established treatment strategies and the alignment of preventive measures across age groups.

Together, these findings underscore the importance of targeted clinical awareness and structured protocols for the management of intoxicated adolescent trauma patients, particularly with regard to airway protection, early detection of complications, and appropriate triage.

## Limitations

This study has several limitations inherent to its retrospective design, which precludes causal inference and introduces potential bias. BAC were not consistently measured, even among severely injured patients, possibly leading to an underestimation of alcohol-intoxicated cases. Moreover, the absence of standardized indications or protocols for BAC testing may have introduced selection bias. Importantly, no data were available on the presence of other substances, such as illicit drugs. This highlights the need for systematic screening of blood alcohol levels in adolescent trauma patients upon emergency department admission. Additionally, dividing the cohort into three age subgroups resulted in limited sample sizes in certain categories, particularly among 10- to 14-year-olds, which may reduce statistical power and introduce bias. Furthermore, as the registry primarily captures prehospital and in-hospital data, it does not provide information on long-term neurological outcomes or functional recovery, limiting conclusions regarding the lasting impact of traumatic brain injury or sepsis observed in the BAL + cohort.

## Data Availability

All data supporting the findings of this study are available within the paper.
